# Editorial Retirement

**Published:** 1894-02

**Authors:** 


### Editorial Retirement.

In the *Dental Review*, Chicago, of January 16th, we learn that Dr. A. W. Harlan retires from the editorial management of the journal.

Dr. Harlan has had editorial charge of the *Review* from its beginning, and through its pages has done much for the profession, and succeeded in establishing a periodical of which he may well be proud. While it is a matter of regret to lose Dr. Harlan from the editorial fraternity, it will be a matter of gratification to not only the readers of the *Review* but to all who know Dr. C. N. Johnson that he is to assume editorial charge, having as associates Drs. T. L. Gilmer and George J. Dennis, and no doubt with these at the helm the *Review* will sustain the position to which it has already attained. We heartily welcome the new editors to the fraternity.

Dr. Harlan left for Europe about two weeks ago, for rest and recuperation. We trust he will ere long return having attained both these conditions in large measure.

